# Retraction: Associations of vitamin D receptor polymorphisms with risk of Alzheimer's disease, Parkinson's disease, and mild cognitive impairment: a systematic review and meta-analysis

**DOI:** 10.3389/fnagi.2025.1557340

**Published:** 2025-01-14

**Authors:** Frontiers Editorial Office

The journal retracts the 12^th^ April 2024 article cited above.

Following publication, concerns were raised regarding the scientific validity of the article. Specifically, ambiguities were introduced by renaming the alleles under study.

An investigation was conducted in accordance with Frontiers' policies.

It was found that the complaints were valid and that the article does not meet the standards of editorial and scientific soundness for Aging Neuroscience; therefore, the article has been retracted.

This retraction was approved by the Chief Editors of Aging Neuroscience and the Chief Executive Editor of Frontiers. The authors do not agree to this retraction.

